# Topics of the Month

**Published:** 1895-11

**Authors:** 


### TOPICS OF THE MONTH.

Mr. H. H. Littell, the optimistic manager of the street railway company, has been abroad. He comes home loaded with information, which he has unloaded to a reporter of the *Express*. After staying four months in Europe, Mr. Littell comes back with nothing but admiration for his own system, which we are constrained to affirm is about the worst in any large city in the Union. Mr. Littell, if he is quoted correctly, affirms that he found but two improvements, while he was abroad, over the Buffalo system, and these were patience and forbearance on the part of the general public.

Every American who knows anything of foreigners is aware that an Englishman is anything but patient and forbearing if he does not get the seat in a tramcar or omnibus that he pays for. In Europe everybody is provided with a seat. In Buffalo this is exceptional. Mr. Littell had better go again and see if he cannot learn something new that is at least truthful to tell us. If, as reported, he owns a controlling interest in the Buffalo street railway system, he had better make haste to remedy the scandalous overcrowding of street cars that is witnessed daily on our stieets. Besides the discomfort growing out of this faulty management it is very unhealthful, and on this ground we shall have occasion to challenge the faults of Mr. Littell's system in future unless they are corrected. Physicians are deeply interested in this subject on sanitary grounds, and it furnishes a wholesome theme for discussion in our medical societies.

---

IN COMPLIANCE with repeated requests, the regents have opened an office in New York City for the accommodation of the immense amount of business connected with the university that comes from the metropolis.

Mr. Asa O. Gallup, formerly chief clerk in the regent's office at Albany, has been appointed to take charge of the New York office. He will have all publications, blanks and necessary records for the accommodation of law, medical, dental and veterinary students, and for all the professional, academic and higher examinations conducted by the university.

The New York office is located at 10 East 42d street, and will be found open during the school week from 9 A. M. to 4 P. M. and 7 to 9 P. M. Business hours will be from 9 A. M. to 12 M., but the deputy will see all callers at other hours if they cannot come between 9 A. M. and 12 M.

---

MR. CHARLES R. SKINNER, superintendent of public instruction (*Buffalo Express*), has begun a campaign for the repeal of that absurd law requiring that pupils in the public schools shall be taught, for at least ten weeks in every school year, the effect of alcohol and narcotics on the human body. Here's more power to his elbow. The instruction which is implied by the law is all right. The method of the instruction and the presumed purpose of the law are all wrong.

This subject will be found fully set forth in its proper light in Dr. Hopkins's article published in this issue of the JOURNAL.

---

THE people's baths of the New York Association for Improving the Condition of the Poor, at No. 9 Centre Market Place, are becoming more popular every year. The total number of baths taken since the opening, on August 17, 1891, is 307,844. Much encouragement is derived by the society in the prosecution of its work from the fact that the number of baths taken last year was 88,734, an advance of 8,197 over the number taken the year before. The volume of business has been immense, in view of the small number of bathtubs at the establishment.

